# Longitudinal prospective cohort study evaluating prognosis in idiopathic intracranial hypertension patients with and without comorbid polycystic ovarian syndrome

**DOI:** 10.1038/s41433-023-02569-x

**Published:** 2023-05-24

**Authors:** Mark Thaller, Victoria Homer, Matilde Sassani, Susan P. Mollan, Alexandra J. Sinclair

**Affiliations:** 1 Translational Brain Science, Institute of Metabolism and Systems Research, University of Birmingham, Edgbaston, Birmingham, B15 2TT, UK; 2 Department of Neurology, University Hospitals Birmingham NHS Foundation Trust, Birmingham, B15 2TH, UK; 3 Centre for Endocrinology, Diabetes and Metabolism, Birmingham Health Partners, Birmingham, B15 2TH, UK; 4 Cancer Research (UK) Clinical Trials Unit, University of Birmingham, Birmingham, B15 2TT, UK; 5 Birmingham Neuro-Ophthalmology, University Hospitals Birmingham NHS Foundation Trust, Birmingham, B15 2TH, UK

**Keywords:** Prognosis, Optic nerve diseases, Outcomes research

## Abstract

**Introduction:**

Idiopathic intracranial hypertension (IIH) and polycystic ovary syndrome (PCOS) are hyperandrogenic metabolic disorders that affect women of reproductive age living with obesity. The previously reported prevalence of comorbid PCOS in IIH patients is highly variable and the longitudinal impact on visual and headache outcomes are unknown.

**Methods:**

In this prospective longitudinal cohort study patients were identified from the IIH: Life database over a nine-year period (2012–2021). Data collected included demographics and PCOS questionnaire data. Key visual and detailed headache outcomes were recorded. We analysed the key variables for influential outcomes of vision and headache. Logistical regression methods were used to model long term visual and headache outcomes.

**Results:**

Overall 398 women with IIH and documented PCOS questionnaires were followed up for a median of 10 months (range 0–87). Prevalence of PCOS in IIH was 20% (78/398) diagnosed by the Rotterdam criteria. Patients with IIH and comorbid PCOS reported higher self-reported fertility problems (3.2-fold increased risk) and increased need for medical help in becoming pregnant (4.4-fold increased risk). Comorbid PCOS in IIH patients does not adversely impact long-term vision or headache outcomes. The headache burden was high in both cohorts studied.

**Conclusions:**

The study demonstrated that comorbid PCOS in IIH is common (20%). Diagnosing comorbid PCOS is important as it can impact on fertility and is known to have long-term adverse cardiovascular risks. Our data suggest that a diagnosis of PCOS in those with IIH does not significantly exacerbate long-term vision or headache prognosis.

## Introduction

Idiopathic Intracranial Hypertension (IIH) is a disease of raised intracranial pressure (ICP) diagnosed by the modified Dandy criteria which typically affects women of reproductive age with obesity [1–3]. The condition causes papilloedema, with a consequent risk of visual loss and chronic disabling headaches [2, 4].

Polycystic ovarian syndrome (PCOS) occurs in women living with obesity of childbearing age which is of a similar phenotype to that observed in IIH. PCOS can be diagnosed by the Rotterdam criteria [5]. The Rotterdam criteria requires at least 2 of the 3 features of menstrual irregularities (oligo-/ anovulation), hyperandrogenism (manifested as hirsutism, acne and alopecia, or biochemical signs), and multiple ovarian cysts (≥12 follicles in each ovary measuring 2–9 mm in diameter, ± ovarian volume >10 ml), with the exclusion of other causes [5]. The NIH criteria require both menstrual and hyperandrogenism features to be present, but not polycystic ovaries [6].

There are several major metabolic features that are common to IIH and PCOS. They are both hyperandrogenic disorders, although characterised by distinct hormonal signatures: androstenedione is predominantly elevated in PCOS, whereas testosterone in IIH [7]. Truncal adiposity and insulin resistance are features of both diseases which also share alterations in adipose tissue functions driving lipogenesis in excess to that attributable to obesity alone [8–11]. In addition, both conditions are associated with an increased risk of cardiovascular disease [12, 13], cognitive impairment [14–16], and obstructive sleep apnoea [17, 18].

PCOS has an established relationship to adverse reproductive health [19–21] and recently this has been reported in IIH where reduced fertility was noted as well as increased risk of pre-eclampsia and gestational diabetes [22].

Thus, despite IIH and PCOS being distinct clinical entities with differential profiles of androgenic steroid dysregulation, they share numerous similar disease manifestations [7]. They have also been observed to coexist, although the exact prevalence of comorbid PCOS in IIH has not clearly been established, as published results are highly variable, ranging from 15% to 57% of IIH patients having concomitant PCOS [23–25].

We hypothesised that in patients with coexisting IIH and PCOS, the metabolic phenotype could be exacerbated, leading to increased morbidity. Amongst patients with IIH, we aimed to evaluate the impact of comorbid PCOS on fertility, as well as the visual and headache prognosis over time.

## Methods

### Study type

A prospective longitudinal observational cohort study was conducted, nested within the IIH: Life study. Data was prospectively collected from consecutive IIH patients attending a specialist IIH clinic at the University Hospitals Birmingham (UHB) NHS Foundation Trust between 2012 and 2021. Data from every sequential visit was recorded and informed consent obtained. The study was ethically approved by NHS National Research Ethics Committee (14/LO/1208), IIH: Life study.

### Eligibility

Patients eligible for inclusion were those with a confirmed diagnosis of IIH as per the modified Dandy criteria [26]. In addition only those consenting to complete the self-reported PCOS questionnaire were eligible (Appendix 1). Those referred with a potential diagnosis of IIH but in whom the diagnosis was not confirmed, those diagnosed with a secondary cause of intracranial hypertension, men and patients with IIH without papilloedema were excluded.

### Diagnosis of PCOS

**Fig. 1:**
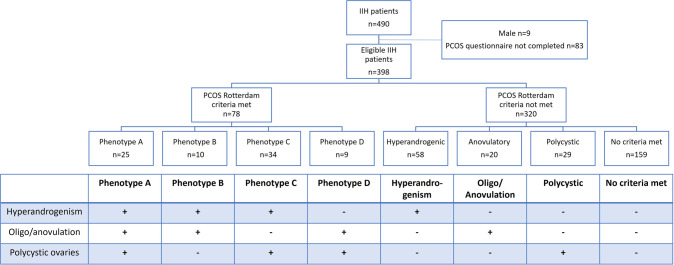
Consort diagram for IIH cohort. The eligible patients were divided by which corresponding features of PCOS by the Rotterdam criteria were present (+) or absent (-).

PCOS diagnosis was established using the Rotterdam criteria [5] thus capturing those with existing confirmed PCOS and those unaware of the diagnosis. The Rotterdam criteria for diagnosis of PCOS require two of the three following criteria after exclusion of other aetiologies: oligo/anovulation, biochemical or clinical signs of hyperandrogenism, and polycystic ovaries (i.e., ≥12 follicles 2–9 mm diameter) in each ovary and/or ovarian volume >10 ml) [5]. Those meeting Rotterdam criteria, were further subdivided into four PCOS phenotypes: A) All three criteria met; B) with hyperandrogenism and oligo/anovulation; C) with hyperandrogenism and polycystic ovaries; D) with oligo/anovulation and polycystic ovaries (Fig. 1) [21]. A diagnosis of PCOS by the Rotterdam criteria was used throughout the analyses to define the PCOS cohort. In addition, we reported descriptive statistics utilising the NIH criteria for PCOS (which comprise a combination of phenotypes A and B) [6]. These are, which was the original diagnostic criteria and are now superseded, but are included in this study to allow comparison with data from historical studies.

### Data collection

Patients were enroled at their first visit to the neuro-ophthalmology clinic. We collected information on patient demographics and the following: body mass index (BMI), body weight, diagnostic lumbar puncture opening pressure, time from diagnostic lumbar puncture to baseline visit (as a surrogate for disease duration), and whether any surgical intervention for IIH was performed. A PCOS questionnaire was completed (Appendix 1) as was a maternal health questionnaire (Appendix 2).

Visual outcomes were assessed by visual acuity (VA) through the logarithm of the minimum angle of resolution (LogMAR), visual field by perimetric mean deviation (PMD, using a Humphrey visual field 24-2 SITA standard) and optical coherence tomography (OCT) imaging (Heidelberg Spectralis™) measurements including global peripapillary retinal nerve fibre layer thickness (RNFL), total retinal thickness (TRT) and macular ganglion cell layer volume (1, 2.22, 3.45 mm volume scan, by either/both of macular volume and posterior pole methods). TRT was calculated by peripapillary cross-sectional imaging using manual segmentation to the basement membrane. This has been reported to be a potentially more accurate measure as automated segmentation in moderate to severe papilloedema using the proprietary software can be inaccurate [27].

Headache outcomes were assessed by headache frequency (number of days per month), migraine-like headache days (number of days per month), headache severity (0-10 numerical rating pain scale, where 0 is no pain to 10 the most severe), and headache disability using the headache impact test-6 (HIT-6) test [28]. We also collected information on medication overuse, previous migraine history, familial history of migraine, and presence of daily headache (defined as headache days of ≥28 days/month) at patient’s first visit to the specialist clinic [29].

### Statistical analysis

Analysis was performed using R (v4.1.0) (https://www.R-project.org/). Summary statistics are presented as mean and standard deviation (SD) for continuous variables, and numbers and percentages for binary or categorical variables.

For visual outcomes (VA, PMD, RNFL, TRT and GCL), the effects of having a PCOS diagnosis versus not were analysed. Data for both eyes were used. Further analyses then investigated important prognostic factors for each outcome, these included the effect of disease duration, the diagnostic lumbar puncture opening pressure (both continuously and categorised <25, 25–29.9,30–39.9, or ≥ 40 cmCSF), BMI at first visit, BMI at each visit, total bodyweight at first visit, total bodyweight at each visit, and baseline RNFL (for non-RNFL outcomes). Models were developed independently for each of the visual outcomes using forward stepwise regression, with our null models adjusting for PCOS as our a priori hypothesis was that this factor was likely to have the greatest effect on outcomes.

For headache outcomes (headache frequency, migraine frequency, headache severity, and HIT-6), the effect of PCOS diagnosis was explored. Further analyses then explored important factors for each outcome exploring the effect of the same covariates as listed under visual outcomes in addition to personal migraine history and daily headache at registration (defined as headache days ≥28 days/month). Models were developed independently for each headache outcome. Comparable processes and models to those employed for visual outcomes were used for headache outcomes, with independent models developed for each headache outcome.

Locally weighted scatterplot smoothing (LOESS) graphs were created prior to regression analysis to ascertain any nascent trends and the association between variables. Regression models were fitted using lme4 [30], with an assuming that dependent variables were continuous. Average response values, an adjustment for time from registration to IIH: LIFE, and an interaction between variables of interest and time-point were estimated using population-level terms. As serial correlation in responses and the nesting of measurements from both eyes occurred, we included patient-level intercepts to address this. There were no diagnostic problems in model fitting. Covariates were transformed or centred around the median value as appropriate when added to models. Independent models were developed for each outcome. There was no imputation for missing data.

### Patient and public involvement

All patients consented for anonymised use of their clinical data, as well as the additional questionnaire data, to be collected and analysed through the IIH: Life database. IIHUK, a national patient charity (Registered Charity in England and Wales no 1143522 & Scotland SCO43294) that supports carers and patients with IIH, endorsed and helped develop the IIH Life concept and questionnaires.

## Results

Overall 490 patients with a confirmed diagnosis of IIH were recruited. Of these 9 were male and 83 did not elect to complete the PCOS questionnaire and were therefore excluded. A confirmed diagnosis of PCOS was made in 78/398 (20%) according to the Rotterdam criteria, whereas 35/398 (9%) met the NIH Criteria (Table 1). Isolated symptomatic hyperandrogenism occurred in 58/398 (15%), oligo/anovulation in 20/398 (5%) and polycystic ovaries in 29/398 (7%) patients (Fig. 1). For the remainder of the analysis, whenever a diagnosis of PCOS is stated, this is by Rotterdam criteria.

**Table 1:** Baseline table by PCOS diagnosis.

	All	PCOS Rotterdam		PCOS NIH	
		Yes	No	Yes	No
IIH patients (*n*)	398	78	320	35	363
Surgical intervention (*n*)	63	20	43	11	52
Age (Mean (SD)) (years)	31.1 (8.5)	30.5 (7.8)	31.2 (8.7)	30.7 (9.4)	31.1 (8.4)
Body mass index (Mean (SD)) (kg/m^2^)	38.9 (8.9)	39.9 (9.5)	38.6 (8.8)	40.8 (9.9)	38.7 (8.8)
Diagnostic lumbar puncture opening pressure (Mean (SD)) (cmCSF)	35.3 (8.2)	35.3 (7.0)	35.3 (8.4)	36.7 (6.9)	35.1 (8.3)

Questionnaire data had missing data fields in 115 patients, however, 9 still met 2 of 3 diagnostic criteria (hyperandrogenism and polycystic ovaries). The most common missing data point being menstrual irregularity (*n* = 102), polycystic ovaries (*n* = 15), and hyperandrogenism (*n* = 13).

BMI at the baseline visit was not significantly higher in the IIH patients with comorbid PCOS compared with those with IIH alone (39.9 (SD 9.5) and 38.6 (SD 8.8), *p* = 0.25) (Table 1). Analogously, there were no significant differences in diagnostic lumbar puncture opening pressures readings between the cohorts (35.3 (SD 7.0) and 35.3 (SD 8.4), respectively, *p* > 0.99) (Table 1).

### Maternal health questionnaire

Data from the maternal health questionnaire, demonstrated that IIH patients with comorbid PCOS were more likely, than IIH patients without PCOS, to report difficulties conceiving (35/71 (49%) versus 43/279 (15%), respectively, *p* < 0.0001). IIH patients with comorbid PCOS were also more likely than IIH patients without PCOS to requiring medical assistance to conceive (14/70 (20%) versus 13/288 (5%), respectively, *p* < 0.001). Amongst the IIH patents with PCOS, there was a 3.2-fold increased risk for self-reported fertility issues and 4.4-fold increase of requiring medical assistance to conceive compared with those with IIH alone.

### Vision measures

At baseline IIH patients with and without PCOS had similar visual acuity (LogMAR), visual fields (PMD), OCT measures of papilloedema (RNFL and TRT) and ganglion cell layer volume (Table 2, Appendix 3).

**Table 2:** Baseline and trajectory (change per month) for visual and headache outcomes by PCOS diagnosis through regression modelling.

	Baseline estimate (units)	Change per month (units/month)
LogMAR visual acuity, logunits		
PCOS	0.0704 (95% CI: 0.0027, 0.138)	−0.0019 (95% CI: −0.0043, 0.0005)
No PCOS	0.0109 (95% CI: −0.0197, 0.0415)	−0.0012 (95% CI: −0.0025, 0.0001)
**Humphrey visual field perimetric mean deviation, dB**		
PCOS	−2.88 (95% CI: −4.8, −0.96)	0.06 (95% CI: −0.05, 0.17)
No PCOS	−3.47 (95% CI: −4.29, −2.65)	0.06 (95% CI: 0.02, 0.1)
**Global peripapillary retinal nerve fibre layer, µm**		
PCOS	141.77 (95% CI: 120.36, 163.18)	−1.44 (95% CI: −2.67, −0.2)
No PCOS	137.76 (95% CI: 128.19, 147.33)	−1.34 (95% CI: −1.99, −0.69)
**Global peripapillary total retinal thickness, µm**		
PCOS	372.33 (95% CI: 342.33, 402.34)	−2.53 (95% CI: −3.95, −1.12)
No PCOS	366.15 (95% CI: 352.74, 379.56)	−2.22 (95% CI: −2.99, −1.46)
**Macular ganglion cell layer volume, mm**^**3**^		
PCOS	0.446 (95% CI: 0.4252, 0.4668)	−0.0006 (95% CI: −0.0009, −0.0002)
No PCOS	0.438 (95% CI: 0.429, 0.447)	−0.0004 (95% CI: −0.0006, −0.0002)
**Headache frequency, days/month**		
PCOS	21.04 (95% CI: 16.68, 25.4)	−0.28 (95% CI: −0.5, −0.06)
No PCOS	18.45 (95% CI: 16.17, 20.74)	−0.11 (95% CI: −0.25, 0.03)
**Migraine-like headache frequency, days/month**		
PCOS	10.22 (95% CI: 6.51, 13.93)	−0.12 (95% CI: −0.29, 0.05)
No PCOS	8.81 (95% CI: 6.78, 10.84)	−0.09 (95% CI: −0.21, 0.03)
**Headache severity, VAS 0**−**10**		
PCOS	6.6 (95% CI: 5.52, 7.69)	−0.0077 (95% CI: −0.0511, 0.0357)
No PCOS	6.43 (95% CI: 5.82, 7.03)	−0.0039 (95% CI: −0.0722, 0.0643)
**HIT-6, score 36**−**78**		
PCOS	59.3 (95% CI: 55.6, 63.01)	0.07 (95% CI: −0.19, 0.33)
No PCOS	61.22 (95% CI: 58.69, 63.75)	0.01 (95% CI: −0.17, 0.18)

**Fig. 2:**
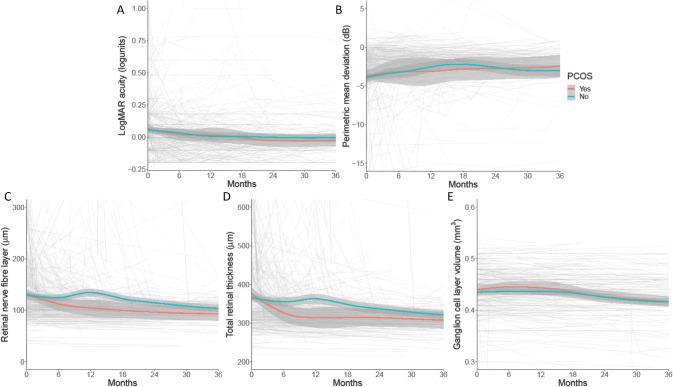
Longitudinal visual data from IIH patients categorised by PCOS diagnosis by Rotterdam criteria, and LOESS smoothers added to show trends across the categories. **A** LogMAR visual acuity (logunits). **B** Perimetric mean deviation measured by Humphrey visual field 24-2 testing (dB). **C** Retinal nerve fibre layer thickness measured on Optical Coherence Tomography (µm). **D** Total retinal thickness of optic nerve head measured on Optical Coherence Tomography (µm). **E** Macular ganglion cell layer volume measured on Optical Coherence Tomography (mm^3^).

The visual outcomes for the IIH cohort with or without comorbid PCOS were then evaluated (Table 2, Fig. 2). Visual acuity outcomes were similar between groups and demonstrated no significant changes over time (Fig. 2A). PMD was similar between the groups showing a gradual improvement following diagnosis and plateauing after ~18 months (Fig. 2B).

Papilloedema measured by OCT analysis of the RNFL and TRT improved more rapidly in those with PCOS (RNFL, −0.134 µm/month (95% CI: −1.524, 1.255) and TRT, −0.315 µm/month (95% CI: 1.916, 1.286)) although this was not statistically significant (Fig. 2C, Appendix 3). In the first 12 months from baseline visit, those patients with comorbid PCOS showed a 20% improvement in TRT (−87.96 µm (SD 99.23)) compared with those without PCOS + 1.25% (+4.57 µm (SD 42.85)), but the difference was not statistically significant (Fig. 2D). Overall, the longer-term papilloedema outcomes were not significantly different in the IIH compared with the IIH with PCOS cohort. Macular ganglion cell layer volume did not differ between groups either at baseline or over longitudinal follow up (Fig. 2E). Similar trends were seen when assessing patients who met NIH criteria (Appendix 4).

### Factors affecting visual prognosis

The factors affecting long-term visual prognosis in IIH alone and IIH with comorbid PCOS were then evaluated. Papilloedema was significantly influenced by the duration of disease prior to the baseline visit (RNFL −0.34 µm/month (95%CI: −0.559, −0.118) and TRT −0.55 µm/month (95%CI: −0.86, −0.25) whilst lumbar puncture opening pressure, BMI and total bodyweight did not. Of these factors only the duration of disease influenced the long-term LogMAR acuity (0.0008 logunits/month (95%CI: 0.0001, 0.0015)). None of these factors impacted the long-term prognosis for visual field PMD although disease duration and first BMI trended towards a significant relationship. Likewise for macular ganglion cell layer volume there was a trend toward significance for total bodyweight.

### Headache measures

**Fig. 3:**
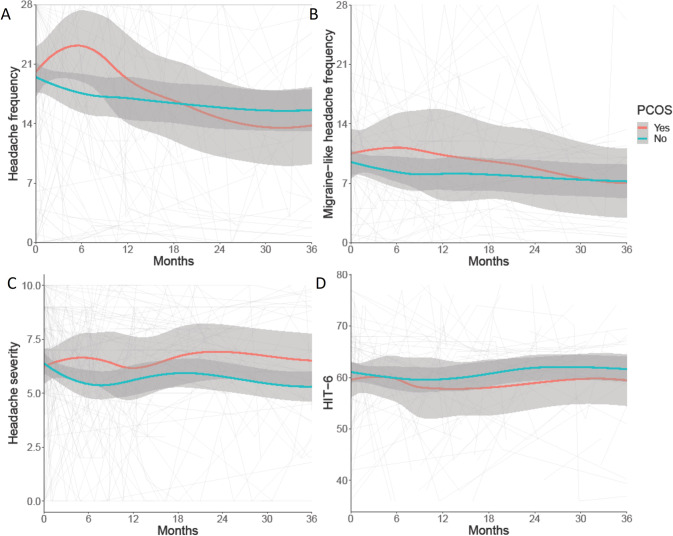
Longitudinal headache data from IIH patients categorised by PCOS diagnosis by Rotterdam criteria, and LOESS smoothers added to show trends across the categories. **A** Headache frequency (days per month). **B** Migraine-like headache frequency (days per month). **C** Headache mean severity of predominant headache (0–10 numerical rating scale). **D** Headache Impact Test 6 (HIT6) (quality of life measure score 36–78).

At baseline both the IIH and the IIH with comorbid PCOS cohorts demonstrated high headache morbidity (headache frequency, migraine-like headache frequency, headache severity, and quality of life (HIT-6)) (Table 2, Fig. 3A–D).

Headache outcomes (headache frequency, migraine-like headache frequency, headache severity, and HIT-6 score) show marked variability and no statistically significant difference between cohorts at baseline or over the period of longitudinal follow up (Table 2, Fig. 3).

Interestingly, headache frequency was observed to improve more rapidly in the PCOS cohort, after an initial peak at 6 months (Fig. 3A). In the IIH cohort with comorbid PCOS, the headache frequency improved by 14% (−2.82 days/month (SD 18.16)) at 12 months and 54% (−10.87 days/month (SD 16.82)) at 24 months. This is in contrast to the IIH cohort without PCOS where headache frequency improved by 4% (−0.65 days/month (SD 9.17)) at 12 months and 24% (−4.28 days/month (SD 9.78)) at 24 months.

### Factors affecting headache outcome

The factors influencing long-term headache prognosis were then evaluated. The only factor influencing long-term headache prognosis in both the IIH and IIH with PCOS cohorts was the occurrence of daily headache at baseline. Personal migraine history, family history of migraine, disease duration, lumbar puncture, BMI, and total bodyweight did not significantly impact headache prognosis in either cohort.

## Discussion

Whilst the comorbid occurrence of PCOS in IIH patients is well known, the clinical impact of a PCOS diagnosis in those with IIH has not yet been evaluated. This is the first prospective longitudinal study of IIH patients with PCOS compared with those with IIH alone. The study provides two key messages. Patients with IIH and PCOS report higher infertility. Comorbid PCOS in IIH patients does not adversely impact long-term vision or headache prognosis.

IIH has been shown to be associated with decreased fertility [22] and infertility has been established in PCOS (although prevalence is highly variable, as it is in the general population) [20, 31–33]. This study adds to the literature by illustrating that dual pathology (IIH and PCOS) may be associated with increased fertility difficulties. The causes are likely multifactorial, but it is plausible that hyperandrogenism, insulin resistance and obesity, factors common to both PCOS and IIH may be important contributors [22]. Our results have implications for clinical practice as they suggest that in those with IIH should have PCOS identified as they may benefit from gynaecological or endocrinological specialist management. Discussing fertility with patients may also include that pregnancy does not adversely impact visual outcomes in IIH in those with well-controlled IIH at the time of conception (particularly in those who have resolved papilloedema) [34].

The OCT imaging measures of papilloedema demonstrated gradual recovery over time in IIH [35]. In this study, a comorbid diagnosis of PCOS did not significantly affect visual outcomes in IIH patients. Obesity is common in both IIH and PCOS. In this study, those with IIH and PCOS had similar baseline BMI to those with IIH alone suggesting that PCOS does not exacerbate the obesity phenotype in IIH. Weight loss is the only current disease-modifying therapy for IIH [35–37], and has been shown to reduce papilloedema and ICP [38–40]. Weight loss is also beneficial in PCOS [21, 31, 41]. There is evidence that the addition of metformin in IIH patients with PCOS could enhance weight loss [42], so could be considered. Specialist support to assist weight loss is advised in IIH irrespective of a diagnosis of PCOS [37].

Headache morbidity in the study was high and predominantly migraine-like in character, as previously described [4, 43–46]. Headaches are a dominant driver of reduced quality of life in IIH and appear to be more refractory to preventive treatment than migraine headaches [47]. In addition, medication overuse headache is common in IIH with high use of opiates [48]. Obesity and metabolic syndrome exacerbate migraine headaches and hence we hypothesised that co-existing PCOS in IIH patients could exacerbate headache morbidity as a result of compounding the adverse metabolic phenotype. In addition, increased headache burden due to migraine has been noted in patients with PCOS [49, 50]. However, we did not find that PCOS was associated with a worse headache prognosis in IIH patients. The headache morbidity was high in the entire IIH cohort, both with and without PCOS. Headache showed little improvement over time and large variability within the IIH population studied. Ongoing severe headaches in IIH are well established and can continue even after papilloedema and ICP resolve as persistent post-IIH headaches [4]. In these case CGRP monoclonal antibody therapies have shown promise for managing headaches in IIH [45, 46].

This study has also highlighted that a diagnosis of PCOS in IIH patients is common, occurring in 20% using Rotterdam criteria (9% by NIH criteria). This is in keeping with the literature which reported 15% by NIH criteria [23]. It is possible that previous studies reporting up to 57% of comorbid POCS and IIH were an overestimate [24, 25]. Regardless of the discrepancies in published results, the prevalence rate of PCOS in IIH remains higher than that observed in the general population (8-13%) [23, 31, 51]. A recent large population study showed a 1.5-fold increased prevalence of comorbid PCOS in IIH patients over age and BMI-matched controls [48, 52].

As with any study there are limitations. This prospectively collected real-world cohort study was based in a tertiary referral centre and therefore a number of patients were initially assessed at other referring hospitals. However, in all cases, the diagnosis was confirmed at the tertiary referral centre. This contributed to baseline visits occurring after different lengths of disease duration (documented as time from diagnostic lumbar puncture to initial visit in the tertiary referral centre). Time to diagnosis was, however, adjusted for in the regression modelling. Although the study aimed to capture all outcomes for consecutive cases, patient preference meant some data were inevitably missing. As per clinical practice, some patients were lost to follow-up and others discharged (for example due to disease remission or referral to alternative hospitals). We cannot exclude the possibility that the results were influenced by those lost to follow-up, nonetheless, this is a limitation inherent to longitudinal studies of this kind. There was also missing questionnaire data, hence, it cannot be excluded that a few PCOS cases might have not been captured. The use of the PCOS questionnaire was an efficient means to identify as many undiagnosed PCOS cases as possible in this IIH cohort, which is important as PCOS remains undiagnosed in the majority of cases (~69% according to published literature) [32]. Patients did not routinely undergo ovarian ultrasound as this is invasive and instead were reliant on patient reporting however where clinically and symptomatically indicated this would have been done either in the community or through endocrinology. The missing data associated with menstrual irregularity may reflect a reluctance to provide personal information seemingly unrelated to IIH headaches and vision, and this should be addressed in future studies. Pragmatically we have used OCT imaging outcome measures that are available through proprietary software. We did not make account of anatomical differences (such as myopia and hypermetropia) that may alter optic nerve head and retinal measurements due to the size of this cohort. In future studies accounting for these changes and indeed the presence of peripapillary hyperreflective ovoid mass structures would be important as they could influence the relationship with other outcomes. Potential other work that is important would be to consider employing analogous analyses to assess whether concomitant PCOS and IIH have adverse impacts on cardiovascular morbidity, cognitive function, and weight loss methods.

## Conclusion

The study demonstrated that comorbid PCOS in IIH is common (20%) and is associated with higher infertility. Our data suggest that a diagnosis of PCOS in those with IIH does not exacerbate long-term vision or headache prognosis. It remains unknown if PCOS in IIH impacts long-term risk of cardiometabolic complications and weight management.

## Summary

### What was known before

- Increased prevalence of PCOS in IIH cohorts, but highly variable in the literature.
- Fertility affected in PCOS and potentially in IIH.

### What this study adds

- Prevalence of comorbid PCOS in IIH is 20% by Rotterdam criteria.
- Self-reported fertility problems were raised in IIH with comorbid PCOS.
- Comorbid PCOS does not exacerbate long-term visual and headache outcomes in IIH.

### Supplementary information

PCOS Questionnaire

Maternal Health Questionnaire

Visual outcomes at 0, 12, 24 and 36 months

NIH visual and headache outcomes

## Data Availability

Professor Sinclair takes full responsibility for the data, the analyses and interpretation, and the conduct of the research. She has full access to all the data; and has the right to publish any and all data separate and apart from any sponsor. Proposals for data access should be made to the corresponding author. Reasonable scientifically sound proposals, from appropriately qualified research groups, will provide data beginning 12 months and ending 3 years after publication of this article to researchers whose proposed use of the data is approved by the corresponding author. Requesters will need to sign a data access agreement, which will cover the terms and conditions of the release of data and will include publication requirements, authorship, acknowledgements, and obligations for the responsible use of data.
