# Supplementary material for: Longitudinal prospective cohort study evaluating prognosis in idiopathic intracranial hypertension patients with and without comorbid polycystic ovarian syndrome
Source: Eye (Lond). 2023 May 24;37(17):3621–8. doi: 10.1038/s41433-023-02569-x (PMC10686374; doi:10.1038/s41433-023-02569-x)
Supplement: Supplementary file 1 — PCOS Questionnaire [file 41433_2023_2569_MOESM1_ESM.docx]

**Appendix 1**

**Polycystic ovarian syndrome questionnaire**

Idiopathic intracranial hypertension can be associated with other hormonal conditions. By answering the questions below, it can help guide us as to whether this might be the case. Your medical team may discuss this further with you.

**Menstrual Cycle**

Do you have regular periods? Yes □ No□ N/A on contraception □

If No, do you get less than 9 periods in a year? Yes □ No□

**Body Hair**

Do you have excess body hair? Yes □ No□

**Polycystic ovaries**

Have you been found to have Polycystic ovaries? Yes □ No□

Have you been diagnosed with Polycystic ovary syndrome? Yes □ No□ Unsure □

If you have any questions having filled out the above questionnaire, please ask your medical team.
