# Supplementary material for: Longitudinal prospective cohort study evaluating prognosis in idiopathic intracranial hypertension patients with and without comorbid polycystic ovarian syndrome
Source: Eye (Lond). 2023 May 24;37(17):3621–8. doi: 10.1038/s41433-023-02569-x (PMC10686374; doi:10.1038/s41433-023-02569-x)
Supplement: Supplementary file 2 — Maternal Health Questionnaire [file 41433_2023_2569_MOESM2_ESM.docx]

**Appendix 2**

**Maternal health questionnaire**

**Pregnancy and Fertility**

Number of previous pregnancies

Number of previous miscarries

Have you postponed plans to get pregnant due to IIH? Yes □ No□

Have you had difficulties getting pregnant? Yes □ No□

Have you ever required to medical help to get pregnant? Yes □ No□

Has IIH influenced your decision to breastfeed? Yes □ No□

Has your choice of contraception been affected by IIH? Yes □ No□

If yes, has contraception stopped? Yes □ No□

Has contraception altered? Yes □ No□

Has pregnancy resulted from inadequate contraception due to changes made by your IIH? Yes □ No□
