# Supplementary material for: Longitudinal prospective cohort study evaluating prognosis in idiopathic intracranial hypertension patients with and without comorbid polycystic ovarian syndrome
Source: Eye (Lond). 2023 May 24;37(17):3621–8. doi: 10.1038/s41433-023-02569-x (PMC10686374; doi:10.1038/s41433-023-02569-x)
Supplement: Supplementary file 3 — Visual outcomes at 0, 12, 24 and 36 months [file 41433_2023_2569_MOESM3_ESM.docx]

**Appendix 3**

**Supplementary Visual outcome table**

Supplementary Table - Visual outcomes according to the presence of PCOS by Rotterdam criteria from LOESS smoothers

|  |  | Months | | | | |
| --- | --- | --- | --- | --- | --- | --- |
|  |  | 0 | 12 | 24 | 36 |  |
| **LogMAR visual acuity, logunits (mean (SD), n)** | PCOS | 0.060 (0.124), 64 | -0.008 (0.129), 24 | -0.025 (0.109), 21 | -0.028 (0.124), 16 |  |
|  | No PCOS | 0.055 (0.192), 334 | 0.010 (0.186), 140 | -0.003 (0.149), 86 | -0.008 (0.169), 57 |  |
| **Humphrey visual field perimetric mean deviation, dB (mean (SD), n)** | PCOS | -3.73 (3.52), 67 | -3.04 (3.76), 24 | -2.68 (3.24), 19 | -3.05 (4.12), 12 |  |
|  | No PCOS | -3.92 (3.80), 327 | -2.49 (4.13), 129 | -2.57 (3.25), 82 | -3.43 (3.39), 47 |  |
| **Global peripapillary retinal nerve fibre layer, µm (mean (SD), n)** | PCOS | 131.67 (39.72), 66 | 105.87 (41.18), 26 | 96.65 (34.34), 21 | 94.18 (36.92), 14 |  |
|  | No PCOS | 130.85 (43.19), 312 | 130.63 (42.15), 129 | 113.68 (31.96), 77 | 104.07 (33.21), 48 |  |
| **Global peripapillary total retinal thickness, µm (mean (SD), n)** | PCOS | 377.73 (66.79), 66 | 317.14 (72.80), 27 | 314.55 (57.93), 22 | 308.56 (62.07), 14 |  |
|  | No PCOS | 366.93 (73.56), 310 | 358.61 (71.51), 129 | 336.03 (55.05), 81 | 320.18 (57.01), 49 |  |
| **Macular ganglion cell layer volume, mm^3^ (mean (SD), n)** | PCOS | 0.440 (0.030), 65 | 0.444 (0.031), 25 | 0.428 (0.026), 21 | 0.415 (0.028), 13 |  |
|  | No PCOS | 0.435 (0.038), 303 | 0.437 (0.037), 120 | 0.424 (0.028), 75 | 0.413 (0.030), 48 |  |
