# Supplementary material for: Longitudinal prospective cohort study evaluating prognosis in idiopathic intracranial hypertension patients with and without comorbid polycystic ovarian syndrome
Source: Eye (Lond). 2023 May 24;37(17):3621–8. doi: 10.1038/s41433-023-02569-x (PMC10686374; doi:10.1038/s41433-023-02569-x)
Supplement: Supplementary file 4 — NIH visual and headache outcomes [file 41433_2023_2569_MOESM4_ESM.docx]

**Appendix 4**

**Supplementary NIH criteria table**

*Supplementary table – Visual and headache outcomes by NIH criteria*

|  | **Baseline estimate** | **Change per month** |
| --- | --- | --- |
| LogMAR visual acuity, logunits |  |  |
| Confirmed PCOS by NIH criteria | 0.0429 (95% CI: -0.0475, 0.1334) | -0.0022 LogMAR/month (95% CI: -0.0048, 0.0005) |
| No PCOS by NIH criteria | 0.0184 (95% CI: -0.0111, 0.048) | -0.0011 LogMAR/month (95% CI: -0.0024, 0.0001) |
| Humphrey visual field perimetric mean deviation, dB |  |  |
| Confirmed PCOS by NIH criteria | -3.66 (95% CI: -6.27, -1.04) | 0.06 dB/month (95% CI: 0.02, 0.1) |
| No PCOS by NIH criteria | -3.35 (95% CI: -4.14, -2.57) | 0.07dB/month (95% CI: -0.06, 0.2) |
| Global peripapillary retinal nerve fibre layer, µm |  |  |
| Confirmed PCOS by NIH criteria | 145.28 (95% CI: 116.49, 174.07) | -1.26 µm/months (95% CI: -2.62, 0.1) |
| No PCOS by NIH criteria | 137.76 (95% CI: 128.57, 146.95) | -1.4 µm/months (95% CI: -2.04, -0.76) |
| Global peripapillary total retinal thickness, µm |  |  |
| Confirmed PCOS by NIH criteria | 374.76 (95% CI: 334.18, 415.33) | -2.06 µm/month (95% CI: -3.64, -0.49) |
| No PCOS by NIH criteria | 366.49 (95% CI: 353.63, 379.36) | -2.36 µm/month (95% CI: -3.1, -1.61) |
| Macular ganglion cell layer volume, mm^3^ |  |  |
| Confirmed PCOS by NIH criteria | 0.448 (95% CI: 0.4203, 0.4757) | -0.0005 µm^3^/month (95% CI: -0.0009, -0.0002) |
| No PCOS by NIH criteria | 0.4384 (95% CI: 0.4297, 0.4471) | -0.0004 µm^3^/month (95% CI: -0.0006, -0.0002) |
| Headache frequency, days/month |  |  |
| Confirmed PCOS by NIH criteria | 19.36 (95% CI: 13.62, 25.1) | -0.21 (95% CI: -0.46, 0.04) |
| No PCOS by NIH criteria | 18.96 (95% CI: 16.79, 21.14) | -0.14 days/month (95% CI: -0.27, 0) |
| Migraine-like headache frequency, days/month |  |  |
| Confirmed PCOS by NIH criteria | 7.79 (95% CI: 2.95, 12.63) | -0.05 days/month (95% CI: -0.24, 0.15) |
| No PCOS by NIH criteria | 9.35 (95% CI: 7.43, 11.27) | -0.11 days/month (95% CI: -0.22, 0.01) |
| Headache severity, VAS 0-10 |  |  |
| Confirmed PCOS by NIH criteria | 6.98 (95% CI: 5.45, 8.51) | -0.0171 units/month (95% CI: -0.0974, 0.0633) |
| No PCOS by NIH criteria | 6.4 (95% CI: 5.83, 6.96) | -0.005 units/month (95% CI: -0.0463, 0.0364) |
| HIT-6, score 36-78 |  |  |
| Confirmed PCOS by NIH criteria | 57.95 (95% CI: 52.79, 63.12) | 0.15 units/month (95% CI: -0.14, 0.43) |
| No PCOS by NIH criteria | 61.19 (95% CI: 58.9, 63.48) | -0.01 units/month (95% CI: -0.18, 0.16) |
